# Letter to the Editor: The effects of hyperlipidaemia, hyperbilirubinaemia and haemolysis on tests performed by the Olympus AU 5000 multiple analyser

**DOI:** 10.1155/S1463924689000210

**Published:** 1989

**Authors:** Robert W. Hardy, Eric D. Green, Frank J. Pikul, David P. Silva, John W. Koenig, Mitchell G. Scott

**Affiliations:** 1 Division of Laboratory Medicine Departments of Pathology and Medicine Washington University School of Medicine St. Louis MO 63110 USA; 2 Clinical Chemistry Laboratory Barnes Hospital St. Louis MO 63110 USA


Journal of Automatic Chemistry Vol. 11, No. 2 (March–April 1989), pp. 89–90

### Letter to the Editor: The effects of hyperlipidaemia, hyperbilirubinaemia and haemolysis on tests performed by the Olympus AU 5000 multiple analyser

**Robert W. Hardy, Eric D. Green, Frank J. Pikul and David P. Silva**
*Division of Laboratory Medicine, Departments of Pathology and Medicine, Washington University School of Medicine, St. Louis, MO 63110, USA*

**John W. Koenig**
*Clinical Chemistry Laboratory, Barnes Hospital, St. Louis, MO 63110, USA*

**and Mitchell G. Scott**
*Division of Laboratory Medicine, Departments of Pathology and Medicine, Washington University School of Medicine, St. Louis, MO 63110, USA*

We systematically investigated the effects of hyperlipidaemia, hyperbilirubinaemia and haemolysis on 20 tests performed by the Olympus AU 5000. This brief report should be useful for users of this new analyser or those using similar analytical approaches.

Increasing amounts of Intralipid (Kabivitrum), bilirubin (NBS standard 916) or haemolysate were added to a serum pool of 40 patients, as previously described [1]. Added Intralipid yielded samples with triglyceride levels of 0·8–6·3 g/l, added bilirubin yielded samples having total bilirubin levels of 4–325 mg/l and added haemolysate yielded samples with haemoglobin concentrations of 0–1·58 g/l. All analyses were performed in duplicate using procedures provided by the manufacturer (Olympus Corp., Success, NY, USA).

The amount of each interfering substance causing at least a 10% negative or positive interference is indicated in table 1. In all instances where interference was observed, higher concentrations of the interferent resulted in larger interferences. For instance, the effects of bilirubin on serum creatinine at bilirubin concentrations of 130 and 190 mg/l produced negative interferences of 28% and 38%, respectively. Intralipid is intended to simulate the light scattering caused by lipoproteins. However, the micelles of Intralipid are more homogeneous than those found *in vivo* and this approach may not always produce results comparable to those with patients' samples [1]. Nevertheless, the lipaemic interference could in every instance be eliminated by ultracentrifugation. As the

*Table 1. Bilirubin, haemoglobin and lipaemia interferences of 20 analyses performed by the Olympus AU 5000*

| Analyte* | | Bilirubin† (mg/l) | Haemoglobin† (g/l) | Intralipid dilution‡ |
|---|---|---|---|---|
| Glucose | (1·1 g/l) | 200 (−) | – | 1:10 (+) |
| BUN | (180 mg/l) | – | 8·0 (+) | – |
| Sodium | (135 mmol/l) | – | – | 1:10 (−) |
| Potassium | (4·3 mmol/l) | – | 0·8 (+) | – |
| Chloride | (98 mmol/l) | – | – | 1:10 (−)§ |
| Creatinine | (11 mg/l) | 50 (−)§ | – | – |
| Calcium | (81 mg/l) | – | 4·0 (+) | 1:10 (+) |
| Phosphorus | (33 mg/l) | – | 4·0 (+) | – |
| Magnesium | (1·6 meq/l) | – | 8·0 (+) | 1:10 (+)§ |
| Hydrogencarbonate | (30 mmol/l) | 70 (+)§ | 0·4 (+) | 1:10 (−)§ |
| Total protein | (54 g/l) | – | 4·0 (+) | 1:10 (+) |
| Albumin | (31 g/l) | – | 8·0 (+) | 1:10 (+) |
| Uric acid | (48 mg/l) | 70 (−)§ | 12·0 (−)§ | – |
| Cholesterol | (1·5 g/l) | – | 8·0 (+) | – |
| Bilirubin | (4 mg/l) | ND¶ | 0·2 (−) | 1:10 (+) |
| CPK | (127 IU/l) | – | 2·0 (+) | 1:10 (+) |
| AST | (23 IU/l) | 320 (+) | 0·8 (+) | 1:10 (+)§ |
| ALT | (33 IU/l) | 320 (+) | 4·0 (+) | 1:50 (−) |
| ALP | (98 IU/l) | – | 8·0 (+) | 1:10 (+) |
| LDH | (190 IU/l) | – | 0·2 (+) | 1:10 (+) |

* Concentrations of serum pool prior to any additions are given in parentheses.
† Concentration of interferring substance which causes a 10% negative (−) or positive (+) interference.
‡ Dilution of Intralipid to a ratio of 1 : 10 and yielding measured triglyceride concentrations of 6·3 g/l.
§ Interferences not reported in the manufacturer's literature.
¶ ND = not determined.

0142–0453/89 $3.00 © 1989 Taylor & Francis Ltd.

bilirubin added to the serum pool was unconjugated and as endogenous bilirubin may include conjugated bilirubin, biliverdin and other metabolites, our *in vitro* study may not totally reflect the analytical results obtained *in vivo*. Because visible haemolysis does not occur until the haemoglobin concentration is approximately 0·2 g/l, the effect of haemolysis on some analytes with interference from haemolysis at this concentration could be missed. For this reason we have already changed our total bilirubin method for the AU 5000 [2]. Seven of the interferences that we detected are not described in the manufacturer's literature, emphasizing the importance of independently evaluating potential interferences with any new procedure and/or analyser.

### References

1. Glick, M. R., and Ryder, K. W., in *Interferographs: Users Guide to Interferences in Clinical Chemistry Instruments* (Science Enterprises, Indianapolis, 1987).
2. Scott, M. G., Lau, B. W. C., Koenig, J. W. *et al., Clinical Chemistry, An Improved Total Bilirubin Method for the Olympus AU 5000 That Decreases Interferences by Hemolysis or Azotemia*, Volume 34, p. 1921 (1988).

### Book review

**Automatic Methods Of Analysis**
By M. Valcarcel and M. D. Luque de Castro (Elsevier, Amsterdam 1988). ISBN 044443005.

This book is a very welcome addition to the literature, not the least because it takes one burden off the reviewer. Having written the first book on Laboratory Automation as long ago as 1974, I have many times checked my conscience to update the work, but alas no time has been found. Now Valcarcel and Luque de Castro have provided a good reason not to update the original. In many respects this is a good book, well researched and covering most of the field of automation. Clearly the work is balanced towards the authors own experiences. The chapter on Flow Injection Analyser has significantly more than three times the number of references than the chapter on conventional continuous flow.

The major attention of the book relates to the techniques of sampling, the various philosophies of Automation, Continuous, Discrete or Batch and the final section deals with various aspects of analytical instrumentation, Spectroscopic Electro-analytical Chromatographic Techniques and Titrators. In a final section the authors attempt to outline some applications in the areas of clinical chemistry, pollution monitoring and also process control. All of these make interesting reading, but they are subject areas in themselves which require a book in their own rights. No attempt has been made to show how the approaches described in these chapters can relate to the analytical chemistry approach, which is a pity.

The one criticism that I have of the book is that the important areas of specification, economics and the effects on management, are not covered at all in this book. It is true that they are touched upon briefly, but they are vital to successful automation and they need to be reviewed. These areas no doubt are outside the experience of the authors, but are significant. None the less, the book is a collossal achievement and it should find a good sales potential in academic and industrial establishments.

I congratulate the authors most warmly and will enjoy keeping their book as a ready reference source for many years to come.

